# Relationship between sacral-abdominal wall distance, movement performance, and spinal alignment in osteoporosis: a retrospective study

**DOI:** 10.1186/s12877-024-04865-x

**Published:** 2024-03-12

**Authors:** Takashi Nagai, Makoto Miyagami, Shota Nakamura, Keizo Sakamoto, Koji Ishikawa, Ichiro Okano, Fumihito Kasai, Yoshifumi Kudo, Nobuyuki Kawate

**Affiliations:** 1 Department of Rehabilitation Medicine, Showa University School of Medicine, 1-5-8 Hatanodai, Shinagawa-ku, 142-8666 Tokyo, Japan; 2 Orthopaedic Surgery, Showa University School of Medicine, 1-5-8 Hatanodai, Shinagawa-ku, 142-8666 Tokyo, Japan

**Keywords:** Aging, SAD (sacral–abdominal wall distance), Movement performance, Osteoporosis, Spinal alignment, Waist circumference

## Abstract

**Background:**

Aging is associated with muscle atrophy, as typified by sarcopenia. Loss of abdominal muscle strength can cause abdominal wall laxity. The purpose of this study was to investigate the relationship between the sacral vertebra–abdominal wall distance (SAD) and movement performance using a simple lateral spine X-ray image for measuring the SAD.

**Methods:**

In this retrospective study, we included women aged ≥ 65 years who were attending the outpatient clinic for osteoporosis at our hospital. A total of 287 patients (mean age ± SD, 76.8 ± 7.1 years) with measured SAD were included in the analysis. Patients were divided into two groups based on SAD cutoff (160 mm) and age (75 years), respectively. The patients were examined using the two-foot 20 cm rise test, 3 m Timed Up and Go (TUG) test, two-step test, open-eyed one-leg standing time, and spinal alignment. Normally distributed data are expressed as means (standard deviations) and non-normally distributed data as medians (interquartile range), depending on the results of the Kolmogorov–Smirnov test. Student’s *t*-test and χ2 test were used for between-group comparisons. Regression analysis was performed with SAD as the objective variable. A two-sided *p* < 0.05 was considered statistically significant.

**Results:**

The shorter SAD group performed better in the two-step test, TUG test, and open-eyed one-leg standing time (*p* < 0.001) as well as in the two-foot 20 cm rise test (*p* < 0.01) compared to the longer SAD group. Spinal alignment was better in the shorter SAD group than in the longer SAD group, with a shorter sagittal vertical axis (*p* < 0.001), smaller pelvic tilt (*p* < 0.001), and greater sacral slope (*p* < 0.05).

**Conclusion:**

SAD was associated with posterior pelvic tilt and movement performance parameters. In addition to testing for osteoporosis, movement performance parameters should be evaluated in women with osteoporosis who are aged ≥ 65 and have greater SAD (≥ 160 mm in this study). The SAD is a new assessment method, and further research is required to verify its validity and reproducibility. This is the first attempt to determine how age and SAD affect movement performance in older adults.

## Background

Globally, the number and proportion of people aged 65 and older are increasing [1]. Japan’s aging rate has reached 29.1%, with one in ten people being over the age of 80 [2]. In Japan, the elderly are defined as those aged 65 or older, those aged 65–74 are classified as elderly in the first half of elderly life, and those aged ≥ 75 years as the elderly in the latter half of elderly life. Those who are ≥ 75 years of age are generally considered borderline because they are more likely to develop infectious diseases and have chronic diseases due to the decline of physiological functions, renal functions, low nutrition, and immune functions. The age of 75 years is generally considered to be the boundary of the disease [3]. Aging is associated with muscle atrophy, as typified by sarcopenia [4, 5]. Longitudinal studies on adults aged > 75 years have reported that muscle mass decreases at a rate of 0.64–0.70% per year in women and 0.80–0.98% per year in men [6]. Age-related muscle loss limits the daily activities of the elderly, such as getting up from a chair, lifting heavy loads, and walking, and increases the risk of disability, hospitalization, and death, imposing a considerable burden on society and healthcare systems [5, 7].

Spinal alignment changes with age. Schwab et al. [8] examined several indices using simple lateral spine radiographs: the sagittal vertical axis (SVA), which is the shortest distance between a vertical line through the center of the C7 vertebral body and the upper margin of the posterior wall of the first sacral vertebra; pelvic tilt (PT), which is the angle between a vertical line through the center of the femoral head and the center of the upper margin of S1; and sacral slope (SS), which is the angle between the upper margin of S1 and the ground, were used as indicators of postoperative spinal alignment. Previous studies have reported that muscle strength, 10-m walking speed, and physical balance decrease and the risk of falling increases when spinal sagittal alignment is disrupted [9, 10].

Several tests are used to assess motor instability and movement performance. Grip strength can indicate the amount of movement performance [11]. Grip strength can be used to predict 10-year cognitive function status, and lower grip strength was shown to be associated with decline in cognitive function and increase in risk of falling [12, 13]. Furthermore, grip strength was also related to preoperative and postoperative spinal alignment in lumbar spinal stenosis [14]. Timed Up and Go (TUG) time was designed to measure basic mobility function, and longer TUG time indicates increased number of falls [15, 16]. Hasegawa et al. [17] reported that higher bite strength or better results in single-leg standing time increased with increase in bone density, which in turn was associated with higher occlusal force or better results of one-leg standing time. Sixty seconds of open-eye unipedal standing can also increase bone mineral density because it applies the same amount of weight to the hip joint as walking for approximately 53 min [18]. The two-step test and rising from a step is one of the diagnostic criteria for locomotive syndrome and can be used to investigate lower limb muscle strength and range of motion of the hip, knee, and ankle joints [19, 20].

Nagai et al. [21, 22] measured the sacral vertebra–abdominal wall distance (SAD), which is the shortest distance between the apex of the abdominal muscles and a vertical line passing through the upper edge of the posterior S1 wall, using radiographic images obtained during routine spine practice. SAD is not related to subcutaneous fat thickness and provides an objective measure of the degree of relaxation of the abdominal muscles and intra-abdominal volume. Furthermore, Nagai et al. [21] reported that grip strength decreased and the risk of falling increased in individuals with greater SAD. Since grip strength has been reported to be related to movement performance and spinal alignment, we hypothesized that SAD may also be related to movement performance and spinal alignment.

This study is the first attempt to determine whether SAD is related to movement performance and spinal alignment using SAD measured from simple X-ray images of the lateral spine.

## Methods

### Ethics

This study was conducted in accordance with the Declaration of Helsinki and the ethical guidelines for medical research involving human participants. Details of the research are available on the hospital website under the Disclosure of Information on Clinical Research Conducted at Showa University (Opt-out)” (https://www.showa-u.ac.jp/visitor/medical/clinical_trial/index.html). This was a retrospective study, and the requirement for informed consent was waived by the Showa University Research Ethics Review Board (approval no. 21-194-A).

### Participants

Among the women attending the medical institution for osteoporosis between 2017 and 2022, 314 women with a measured SAD were enrolled. Women were diagnosed with osteoporosis in cases of fragility vertebral fractures or proximal femur fractures, and in those without fragility fractures but with bone mineral density < 70% of the young adult mean value or T-score < -2.5 standard deviations (SDs) [23]. Patients aged < 65 years and those who could not be radiographed in the standing position were excluded.

### Study design

In this retrospective study, participants were divided into two groups: 65–74-years and ≥ 75-years. WHO’s expert panel has set the standard body mass index (BMI) for Asians at 23.0 kg/m^2^ [24]. The standard BMI for Japanese people in order to be less susceptible to disease is considered to be 22.0 [25]. Therefore, we examined the cut-off value of SAD in standard BMI using receiver operating characteristic analysis and further divided the patients into two groups by cut-off value. Finally, patients were divided into four groups based on age and SAD.

### Patients’ general condition

#### Assessment of history and complications

CCI (Charlson Comorbidity Index) was used to investigate patients’ comorbidities: 1 point each, for myocardial infarction, congestive heart failure, peripheral artery disease, dementia, cerebrovascular disease, chronic lung disease, collagen disease, peptic ulcer, mild liver disease, and uncomplicated diabetes; 2 points each, for hemiplegia, moderate to severe hemiplegia, moderate to severe renal disease, diabetes with complications, localized solid tumors, leukemia, and lymphoma; moderate to severe liver disease receives 3 points each; and metastatic solid tumors and AIDS (acquired immunodeficiency syndrome) receive 6 points each [26, 27].

#### Clinical history level

The level of clinical function was assessed using a frailty score. The frailty assessment was performed using a 5-item questionnaire previously described by Yamada et al. [28]. The five items were: (1) Have you lost 2 to 3 kg weight in the last 6 months? (Yes, 1 point), (2) Do you have difficulty crossing a pedestrian crossing at a green light? (Yes, 1 point), (3) Do you take a walk or otherwise exercise at least once a week? (No, 1 point), (4) Do you have difficulty opening plastic bottles or plastic bottle lids? (Yes, 1 point), (5) In the last two weeks, have you felt tired for no reason? (Yes, 1 point).

#### Visual analog scale (VAS)

Low back pain was checked directly with the patients using a VAS just prior to performing the movement performance tests (grip strength, open-eyed one-leg stand, 3-m TUG, and two-step tests).

#### Assessment of risk of fall

To assess fall risk, a fall score consisting of five data items based on activities of daily living was used [29]. Scores for each item were calculated based on odds ratios, and individuals scoring 6 or higher were considered at high risk for falls [13, 29, 30]. If a participant answered “yes,” they were assigned 5 points for the first question and 2 points for each of questions 2–5. Participants with a total score of ≥ 6 were considered at high risk for falls.

### Test items

#### Bone mineral density

Bone mineral density and body composition were measured using dual-energy X-ray absorptiometry (Discovery DXA System; Hologic, Inc., Marlborough, MA, USA). Bone mineral density was measured at the lumbar spine and femoral neck, whereas trunk fat percentage was measured throughout the body.

### Hand grip strength

Grip strength was measured using a Smedley hand dynamometer (MY-2080; Matsumiya Medical Seiki Corporation, Tokyo, Japan). Measurements were performed by a state-licensed physical or occupational therapist. Grip strength was measured three times in a sitting position, based on previous studies [10, 13, 30]. The median value of the three measurements was used as the left and right grip strength, and the better-performing value between the left and right was used as the grip strength.

#### Open-eyed one-leg standing time

The time to stand with one leg was measured by raising one leg to a height of 10 cm for a maximum of 15 s with the eyes open and both hands placed on the waist. The participants were instructed beforehand not to let the raised leg touch the axial leg. If the axis leg moved, the raised leg touched the floor, or the arm moved away from the waist, the time until that point was recorded as the end of the test. The higher value between the left and right leg was used.

#### Standing up

The participants were placed on 10-, 20-, 30-, and 40-cm high wooden platforms and examined based by their ability to stand up without recoil from a sitting position with their hands folded. Because the ability to stand up from a 20-cm platform with both feet is divided into locomotion level 1 and locomotion level 2 [19], standing up from a 20-cm platform was investigated separately.

#### Two steps

The starting line was set, the toes of both feet were placed together, the subject took two steps as large as possible, and the stride length of the two steps with both feet together (from the line where they first stood to the toe of the landing point) was measured. If the patients lost balance, they were instructed to start over; two measurements were taken and the better record was used.

As in the previous study, the distance (m) of the two large steps from rest was measured by dividing the distance (m) by the height (m) [31].

#### 3-m timed up and go test

In this TUG test, participants seated in a chair, got up from the chair at the start signal, walked around a pole 3 m away, and the time it took them to sit in the chair again was measured. Participants were instructed to walk faster than their normal walking speed.

#### Spinal alignment and SAD

**Fig. 1:**
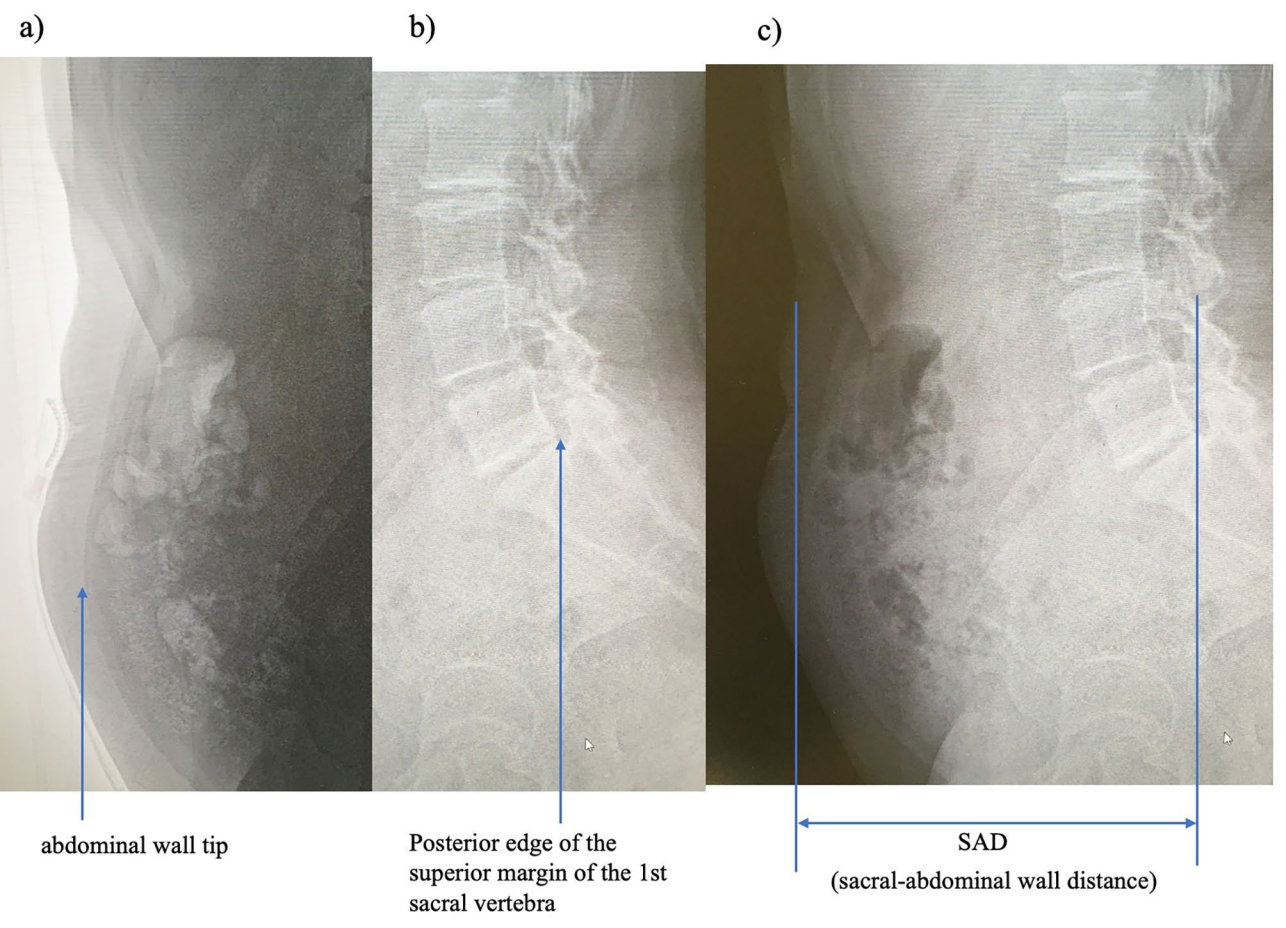
Sacral-abdominal wall distance (SAD). **(a)** Tip of abdominal wall in soft tissue condition, **(b)** Posterior edge of the superior margin of the first sacral vertebra in bone condition, **(c)** SAD is the distance between the tip of the abdominal wall and the posterior edge of the upper margin of the first sacral vertebra.

The SVA, PT (pelvic tilt), SS (sacral slope), and SAD were measured using simple lateral spine radiographs (Medical Systems USA, Inc., version 4.1.50107, New York, NY, USA). If the transition vertebrae caused difficulty in determining the first sacral vertebra, the 25th vertebra from the first cervical vertebra was used as the first sacral vertebra. The patients assumed a natural standing position for simple lateral radiographs of the spine, with their hands touching the shoulders to ensure that the upper extremities and spine did not overlap. During imaging, the patients were asked to inhale and hold their breath. Patients wore an examination gown provided by the hospital before the examination (Fig. 1). The intraclass correlation coefficient was 0.998, and the average error between the two measurements was 1.5 mm.

### Data analysis and statistical methods

In this study, normally distributed data are expressed as means (standard deviations [SD]) and non-normally distributed data as medians (interquartile range), depending on the results of the Kolmogorov–Smirnov test. The Student’s *t*-test and χ2 test were used for between-group comparisons. A two-sided *p*-value < 0.05 was considered statistically significant. The VAS scores were correlated with the VAS using the Pearson Product-Moment Correlation to determine if the VAS score affects the two-step, 3-m TUG, and open-eye one-leg standing times and grip strength. Regression analysis was performed with SAD as the objective variable with age, BMI, waist circumference, CCI, frailty index, femur bone mineral density, Visual Analogue Scale, SVA, and PT as dependent variables. Receiver operating characteristic analysis was used to determine the cutoff value of SAD. Analyses were performed using StatFlex (ver. 7.0.8; Medical Watch Institute, Ube, Japan). To avoid reducing the data (sample) size, missing data were ignored, and no imputation methods were applied.

### Required sample size

A sample size test was performed using G*Power (ver. 3.1.9.6; Heinrich Heine University, Düsseldorf, Germany), with an alpha error of 0.05, a power (1-beta error) of 0.95, and an effect size of 0.5. The required sample size was 210.

## Results

### Baseline patient characteristics and group assignment

**Fig. 2:**
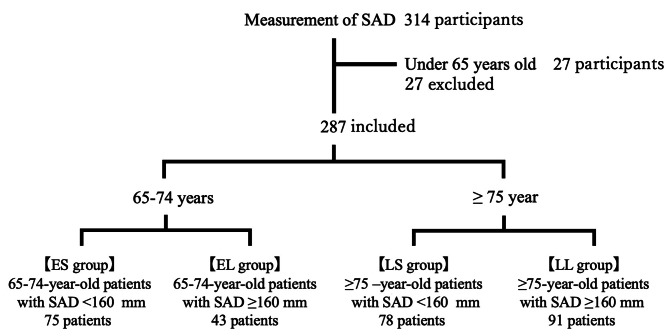
Study protocol. Of the 314 women whose abdominal wall-sacral distance (SAD) was measured, 287 were aged ≥ 65 years and were divided into two groups: 65–74-year and ≥ 75-year group; each group was further divided by a median SAD of 160 mm. ES group: 65–74-year-old patients with SAD < 160 mm; EL group: 65–74-year-old patients with SAD ≥ 160 mm; LS group: ≥75-year-old patients with SAD < 160 mm; LL group: ≥75-year-old patients with SAD ≥ 160 mm.

Among the 314 women enrolled, 27 patients aged < 65 years were excluded. Patients’ baseline characteristics were extracted from the existing data. Data for 287 women aged ≥ 65 years who visited the osteoporosis outpatient clinic and had abdominal wall-sacral distance measurements, were included in the final analysis. The mean age of the 287 participants was 76.8 ± 7.1 years (mean ± SD) (Fig. 2). Patient characteristics are summarized in Table 1. Overall, 121 patients (42.2%) had a bone mineral density <-2.5 SD at the femoral neck.

**Table 1:** Patient characteristics between the 65–74-year group and ≥ 75-year group

	Total	65-74 years	≥ 75 years	*P*-value
Numbers	287	118	169	
Age (years), mean (SD)	76.8 (7.1)	70.1(3.1)	81.6 (4.9)	< 0.001
BMI, mean (SD)	21.6 (3.5)	21.4 (3.5)	21.6 (3.6)	0.64
< 18.5, n (%)	59 (20.6)	20 (16.9)	39 (23.1)	0.13
18.5 − 25, n (%)	188 (65.5)	85 (72.0)	103 (60.9)	
25 − 30, n (%)	35 (12.2)	10 (8.5)	25 (14.8)	
> 30, n (%)	5 (1.7)	3 (2.5)	2 (1.2)	
Height (cm), mean (SD)	150.1 (6.3)	152.2 (6.2)	148.7 (6.0)	< 0.001
Weight (kg), mean (SD)	48.5 (8.1)	49.5 (7.6)	47.8 (8.3)	0.08
Waist circumference (cm), mean (SD)	80.4 (10.0)	79.5 (9.9)	80.9 (10.1)	0.26
Trunk fat (%), mean (SD)	30.2 (8.4)	30.8 (8.1)	29.7(8.6)	0.29
Anti-osteoporotic drugs				
Any, n (%)	269 (93.7)	110 (93.2)	156 (92.3)	0.77
SERM, n (%)	73 (25.4)	42 (35.6)	31(18.3)	< 0.05
Bisphosphonate, n (%)	121 (42.2)	45 (38.1)	31(18.3)	
Denosumab, n (%)	56 (19.5)	17 (14.4)	39 (23.1)	
Teriparatide, n (%)	5 (1.7)	1 (0.8)	4 (2.4)	
Anti-sclerostin Antibody, n (%)	5 (1.7)	1 (0.8)	4 (2.4)	
Fractur any, n (%)	170 (59.2)	49 (41.5)	121 (71.6)	< 0.001
Fractur spine, n (%)	164 (57.1)	46 (39.0)	118 (69.8)	< 0.001
Fractur hip, n (%)	9 (3.1)	3 (2.5)	6 (3.6)	0.63
SAD (mm), mean (SD)	162.4 (25.5)	156.2 (24.1)	166.7 (25.6)	< 0.001
CCI (points), Median [IQR]	0 [0 − 2]	0 [0 − 2]	0 [0 − 2]	0.24
Bone Mineral Density (Spine; g/cm^2^), Median [IQR]	0.789 [0.688 − 0.895]	0.764 [0.676 − 0.875]	0.812 [0.706 − 0.944]	< 0.05
Bone Mineral Density (Hip neck; g/cm^2^), mean (SD)	0.528 (0.093)	0.535 (0.091)	0.524 (0.095)	0.34
Bone Mineral Density (Hip neck; T score), mean (SD)	-2.8 (3.5)	-2.6 (2.1)	-2.9 (4.3)	0.40
Hand grip (kg), mean (SD)	18.8 (4.6)	20.6 (3.6)	17.6 (4.7)	< 0.001
2 Step, Median [IQR]	1.25 [1.09 − 1.37]	1.35 [1.20 − 1.46]	1.18 [0.98 − 1.30]	< 0.001
3 m Timed Up and Go test (s), Median [IQR]	7.2 [6.0 − 9.0]	6.4 [5.5 − 7.6]	8.1 [6.6 − 10.5]	< 0.001
Open eye unipedal standing (s), Median [IQR]	15.0 [4.8 − 15.0]	15.0 [15.0 − 15.0]	7.0 [2.9 − 15.0]	< 0.001
Rise 40 cm clear	37 (12.9)	4 (3.4)	33 (19.5)	< 0.001
Rise 30 cm clear	26 (9.1)	7 (5.9)	19 (11.2)	
Rise 20 cm clear	66 (23.0)	22 (18.6)	44 (26.0)	
Rise 10 cm clear	140 (48.8)	82 (69.5)	58 (34.3)	
Starts up, 40 m not possible.	14 (4.9)	2 (1.7)	12 (7.1)	
Startup unchecked	4 (1.4)	1 (0.8)	3 (1.8)	
VAS (lumber), Median [IQR]	2.0 [0.0 − 3.0]	2.0 [0.0 − 3.0]	2.5 [0.0 − 4.0]	< 0.01
PT (^°^), mean (SD)	21.5 (10.7)	19.1(9.3)	23.2(11.3)	< 0.01
SVA (mm), Median [IQR]	19.3 [-16.9 − 42.4]	29.8 [9.3 − 57.9]	55.0 [29.0 − 93.9]	< 0.001
SS (^°^), mean (SD)	29.4 (10.0)	30.7(8.6)	28.4 (10.7)	< 0.05
Has an exercise habit(%)	155 (54.0)	66 (58.9)	89 (56.3)	0.67
Has had a fall in the past year(%)	61 (21.3)	23 (19.5)	38 (22.5)	0.48
Fall risk (points), mean (SD)	4.7 (3.4)	3.6 (3.1)	5.5 (3.4)	< 0.001

Equivariance is expressed as mean (standard deviation), and a lack of equivariance is expressed as median (interquartile ranges). SD: standard deviation, n: number, IQR: interquartile range, BMI: body mass index, SERM: selective estrogen receptor modulator, CCI: Charlson Comorbidity index, eGFR: estimated glomerular filtration rate, HbA1c: hemoglobin A1c, PT: pelvic tilt, SVA: sagittal vertical axis, SAD: sacral vertebra–abdominal wall distance.

### 65–74-year and ≥ 75-year groups

No significant differences were observed in BMI, waist circumference, or fat percentage in the torso between the 65–74-year group (*n* = 118 participants) and ≥ 75-year group (*n* = 169 participants). The ≥ 75-year group had significantly shorter two-step, 3-m TUG, and rise times than the 65–74-year group (*p* < 0.001). Grip strength and open-eye one-leg standing times were significantly lower in the ≥ 75-year group than in the 65–74-year group (*p* < 0.001). Back pain score was significantly higher in the ≥ 75-year group than in the 65–74-year group (*p* < 0.01). Spinal alignment was significantly greater for the SVA and PT (*p* < 0.01) and significantly lower for the SS (*p* < 0.05) in the ≥ 75-year group than in the 65–74-year group (Table 1).

#### SAD based on standard BMI

The cut-off value for SAD in Asian women with a BMI of 23 [24] as determined by the WHO expert panel is 161.3 mm (sensitivity = 0.69). The cut-off value of SAD at BMI 22 [25], which is considered standard for the Japanese population, is 159.3 mm (sensitivity = 0.72). In this study, the SAD based on the standard BMI was set at 160 mm, an average value of the two abovementioned standard values.

#### Differences based on SAD

Patients were divided into two groups based on a SAD value of 160 mm and according to the cut-off value of standard BMI; participants in the group with SAD < 160 mm were younger with lower BMI than participants in the group with SAD ≥ 160 mm (*p* < 0.001). Regarding movement performance, the group with SAD < 160 mm performed better than that with SAD ≥ 160 mm in grip strength and two-step, 3-m TUG, and open-eye one-leg standing times (Table 2).

**Table 2:** Patient characteristics between the SAD < 160 mm group and SAD ≥ 160 mm group

	Total	SAD < 160 mm	SAD ≥ 160 mm	*P*-value
Numbers	287	153	134	
Age (years), mean (SD)	76.8 (7.1)	75.4(6.6)	78.5 (7.2)	< 0.001
BMI, mean (SD)	21.6 (3.5)	20.1 (2.9)	23.2 (3.4)	< 0.001
< 18.5, n (%)	59 (20.6)	49 (30.1)	10 (7.5)	< 0.001
18.5 − 25, n (%)	188 (65.5)	98 (60.1)	90 (67.2)	
25 − 30, n (%)	35 (12.2)	5 (3.3)	30 (22.4)	
> 30, n (%)	5 (1.7)	1 (0.7)	4 (3.0)	
Height (cm), mean (SD)	150.1 (6.3)	151.5 (5.6)	148.6 (6.8)	< 0.001
Weight (kg), mean (SD)	48.5 (8.1)	46.2 (7.2)	51.2 (8.2)	< 0.001
Waist circumference (cm), mean (SD)	80.4 (10.0)	76.1 (8.6)	85.3 (9.3)	< 0.001
Trunk fat (%), mean (SD)	30.2 (8.4)	28.5 (7.5)	32.1 (9.1)	< 0.001
Anti-osteoporotic drugs				
Any, n (%)	269 (93.7)	145 (94.8)	124 (92.5)	
SERM, n (%)	73 (25.4)	44 (28.8)	29 (21.6)	0.38
Bisphosphonate, n (%)	121 (42.2)	67(43.8)	54 (40.3)	
Denosumab, n (%)	56 (19.5)	26(17.0)	30 (22.4)	
Teriparatide, n (%)	5 (1.7)	2 (1.3)	3 (2.2)	
Anti-sclerostin Antibody, n (%)	5 (1.7)	4 (2.6)	1 (0.7)	
Fractur any, n (%)	170 (59.2)	77 (50.3)	93 (69.4)	
Fractur spine, n (%)	164 (57.1)	73 (47.7)	91 (67.9)	0.19
Fractur hip, n (%)	9 (3.1)	6 (3.9)	3 (2.2)	
SAD (mm), mean (SD)	162.4 (25.5)	143.7 (13.0)	183.8 (18.5)	*P* < 0.001
CCI (points), Median [IQR]	0 [0 − 2]	0 [0 − 2]	0 [0 − 2]	0.43
Frailty Index (points), mean (SD)	1.3 (1.1)	0.9 (0.8)	1.6 (1.2)	< 0.001
Bone Mineral Density (Spine; g/cm^2^), Median [IQR]	0.789 [0.688 − 0.895]	0.753 [0.673 − 0.840]	0.834 [0.710 − 0.953]	< 0.001
Bone Mineral Density (Hip neck; g/cm^2^), mean (SD)	0.528 (0.093)	0.519 (0.087)	0.539 (0.100)	0.07
Hand grip (kg), mean (SD)	18.8 (4.6)	19.5 (4.7)	18.1 (4.3)	< 0.01
2 Step, Median [IQR]	1.25 [1.09 − 1.37]	1.31 [1.16 − 1.44]	1.19 [0.95 − 1.30]	< 0.001
3 m Timed Up and Go test (s), Median [IQR]	7.2 [6.0 − 9.0]	6.4 [5.8 − 7.8]	8.3 [7.0 − 11.1]	< 0.001
Open eye unipedal standing (s), Median [IQR]	15.0 [4.8 − 15.0]	15.0 [8.5 − 15.0]	7.6 [2.4 − 15.0]	< 0.001
Rise 40 cm clear	37 (12.9)	14 (9.2)	23 (17.2)	< 0.01
Rise 30 cm clear	26 (9.1)	7 (4.6)	19 (14.2)	
Rise 20 cm clear	66 (23.0)	30 (19.6)	36 (26.9)	
Rise 10 cm clear	140 (48.8)	99 (64.7)	41 (30.6)	
Starts up, 40 m not possible.	14 (4.9)	0 (0)	14 (10.4)	
Startup unchecked	3 (1.0)	3 (2.0)	1 (0.7)	
VAS (lumber), Median [IQR]	2.0 [0.0 − 3.0]	2.0 [0.0 − 3.0]	3.0 [1.0 − 4.0]	< 0.001
PT (^°^), mean (SD)	21.5 (10.7)	16.5 (8.7)	27.1 (10.0)	< 0.001
SVA (mm), Median [IQR]	19.3 [-16.9 − 42.4]	29.2 [7.7 − 56.1]	64.5 [32.9 − 103.3]	< 0.001
SS (^°^), mean (SD)	29.4 (10.0)	31.6 (8.7)	26.8 (10.7)	< 0.001
Has an exercise habit(%)	155 (54.0)	66 (55.9)	89 (52.7)	< 0.001
Has had a fall in the past year(%)	61(21.3)	29 (19.0)	32 (23.9)	0.27
Fall risk (points), mean (SD)	4.7 (3.4)	3.7 (3.1)	5.9 (3.4)	< 0.001

Equivariance is expressed as mean (standard deviation), and a lack of equivariance is expressed as median (interquartile ranges). SD: standard deviation, n: number, IQR: interquartile range, BMI: body mass index, SERM: selective estrogen receptor modulator, CCI: Charlson Comorbidity index, eGFR: estimated glomerular filtration rate, HbA1c: hemoglobin A1c, PT: pelvic tilt, SVA: sagittal vertical axis, SAD: sacral vertebra–abdominal wall distance.

### 65–74-year and ≥ 75-year groups and SAD

**Fig. 3:**
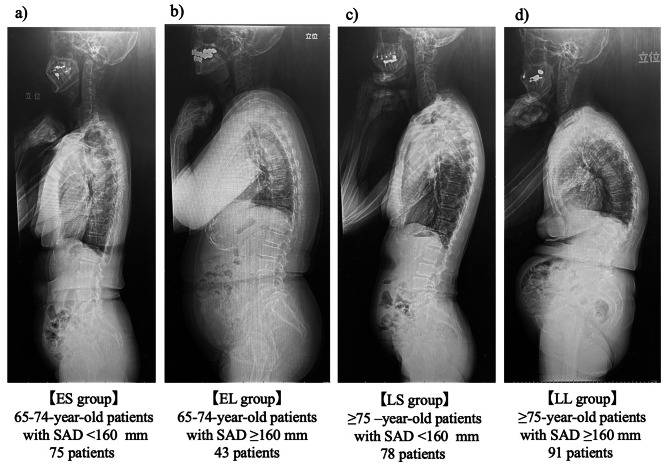
Representative simple X-ray whole spine lateral images and number of patients in each group. **(a)** ES group: 65–74-year-old patients with abdominal wall-sacral distance (SAD) < 160 mm, 75 patients, **(b)** EL group: 65–74-year-old patients with SAD ≥ 160 mm, 43 patients, **(c)** LS group: ≥75-year-old patients with SAD < 160 mm, 78 patients, **(d)** LL group: ≥75-year-old patients with SAD ≥ 160 mm, 91 patients.

Participants in the 65–74-year and ≥ 75-year groups were further divided based on SAD, with 75, 43, 78, and 91 patients in the ES (65-74-year-old patients with SAD < 160 mm), EL (65-74-year-old patients with SAD ≥ 160 mm), LS (≥ 75–year-old patients with SAD < 160 mm), and LL (≥ 75-year-old patients with SAD ≥ 160 mm) groups, respectively (Fig. 3; Table 3).

**Table 3:** The 65–74-year and ≥ 75-year-old patients were classified into four categories based on a median SAD of 160 mm

	[ES]	[EL]	*p*- value	[LS]	[LL]	*p*- value
the number of people	75	43		78	91	
Age (years), mean (SD)	69.8 (3.1)	70.7 (3.0)	0.14	80.8 (4.1)	82.2 (5.4)	0.06
BMI (kg/m^2^), mean (SD)	20.3 (2.90)	23.5 (3.4)	< 0.001	19.2 (2.9)	23.1 (3.5)	< 0.001
Abdominal circumstance (cm), mean (SD)	76.5 (9.0)	84.9 (9.2)	< 0.001	75.7 (8.2)	85.6 (9.4)	< 0.001
CCI (points), Median [IQR]	0 [0 − 2]	0 [0 − 1]	0.62	1 [0 − 2]	0 [0 − 2]	0.38
Frailty Index (points), mean (SD)	0.9 (0.8)	1.0 (0.9)	0.34	1.3 (1.0)	1.8 (1.2)	< 0.01
trunk fat percentage (%), mean (SD)	29.2 (7.6)	33.7 (8.4)	< 0.01	27.8 (7.4)	31.4 (9.4)	< 0.01
2 Step (m/m), Median [IQR]	1.39 [1.28 − 1.49]	1.28 [1.16 − 1.39]	< 0.001	1.22 [1.11 − 1.34]	1.11 [0.89 − 1.25]	< 0.001
3 m TUG (s), Median [IQR]	5.9 [5.3 − 6.9]	7.3 [6.2 − 8.3]	< 0.001	7.0 [6.1 − 8.9]	9.1 [7.3 − 12.4]	< 0.001
Rise 40 cm clear	2 (2.7)	2 (4.7)	< 0.001	12 (15.4)	21 (23.1)	< 0.001
Rise 30 cm clear	1 (1.3)	6 (14.0)		6 (7.7)	13 (14.3)	
Rise 20 cm clear	9 (12.0)	13 (30.2)		21 (26.9)	23 (25.3)	
Rise 10 cm clear	62 (82.7)	20 (46.5)		37 (47.4)	21 (23.1)	
Starts up, 40 m not possible.	0 (0.0)	2 (4.7)		0 (0.0)	12 (13.2)	
Stand up 20 cm with both feet, n (%). Possible	71 (95)	33 (77)	< 0.001	58 (74)	44 (48)	< 0.001
Stand up 20 cm with both feet n (%). Impossible	3 (4)	10 (23)		18 (23)	46 (51)	
Hand Grip (kg), mean (SD)	21.0 (3.5)	19.9 (3.7)	0.12	18.0 (5.2)	17.2 (4.3)	0.14
Unipedal standing (s), Median [IQR]	15.0 [15.0 − 15.0]	15.0 [6.8 − 15.0]	< 0.001	11.4 [4.7 − 15.0]	5.4 [1.9 − 15.0]	0.001
VAS (lumber), Median [IQR]	1.0 [0.0 − 3.0]	2.0 [0.0 − 4.0]	< 0.01	2.0 [0.0 − 3.0]	3.0 [1.0 − 4.0]	< 0.05
SVA (mm), Median [IQR]	18.8 [1.6 − 32.9]	43.2 [31.3 − 77.5]	< 0.001	46.7 [16.8 − 67.6]	73.7 [41.5 − 106.9]	< 0.001
PT (^°^), mean (SD)	15.9 (7.9)	24.7 (9.1)	< 0.001	17.1 (9.4)	28.3 (10.3)	< 0.001
SS (^°^), mean (SD)	32.0 (6.8)	28.5 (10.7)	< 0.05	31.2 (10.2)	26.0 (10.7)	< 0.01
Fractur spine, n (%)	25 (33.3)	21 (48.8)	0.10	48 (61.5)	70 (76.9)	< 0.05
Exercise habit, n (%)	45 (60.0)	21 (48.8)	0.24	40 (51.3)	49 (53.8)	0.74
Has had a fall in the past year(%)	12 (16.0)	11 (26.2)	0.21	17 (22.4)	21 (23.9)	0.84
Fall risk (points), mean (SD)	2.9 (2.8)	4.9 (3.3)	< 0.001	4.5 (3.1)	6.3 (3.3)	< 0.001

ES group, 65-74-year-old with SAD < 160 mm; EL group, 65-74-year-old with SAD ≥ 160 mm;

LS group,75-year-old patients with SAD < 160 mm; LL group, ≥ 75-year-old patients with SAD ≥ 160 mm

SAD: abdominal wall-sacral distance, BMI: body mass index, CCI: Charlson Comorbidity index, TUG: timed up and go, VAS: Visual Analogue Scale, PT: pelvic tilt, SVA: sagittal vertical axis, SS: Sacral slope.

#### Physical findings

No significant differences were noted in age or grip strength between the ES and EL groups or between the LS and LL groups. Waist circumference and trunk fat percentage were significantly greater in groups with higher SAD (the EL and LL) than in groups with lower SA (the ES and LS groups) (Table 3).

#### Movement performance

**Fig. 4:**
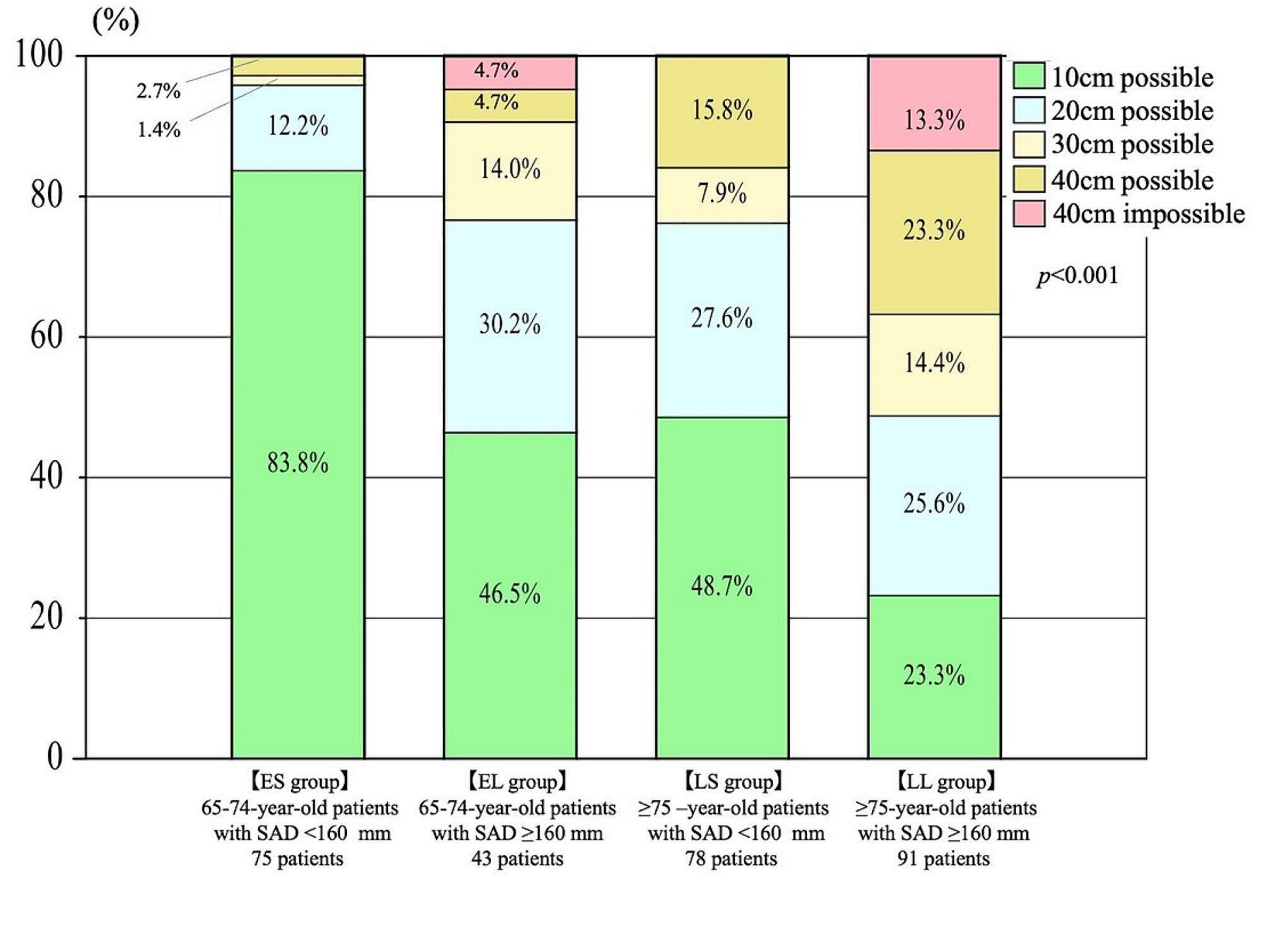
Results of the rise test in the ES, EL, LS, and LL groups. The ES group performed better in the rise from 10 cm, the EL and LS groups did not differ significantly in the rise from 10 to 20 cm, the LL group performed the worst in the rise from 10 cm, and the ES group performed better in the rise from 20 cm than the LS group. ES group, 65–74-year-old patients with SAD < 160 mm; EL group, 65–74-year-old patients with SAD ≥ 160 mm; LS group, ≥ 75-year-old patients with SAD < 160 mm; LL group, ≥ 75-year-old patients with SAD ≥ 160 mm.

Among the participants of the ES and EL groups and the LS and LL groups, the groups with shorter SAD (ES and LS) performed better in the two-step, 3-m TUG, and open-eyed one-leg standing time tests (*p* < 0.001). In the toughest condition (standing from 10 cm), the ES group had the highest percentage of standing at 83.8%, the EL and LS groups had similar percentages at 46.5 and 48.7%, respectively, and the LL group had the lowest percentage at 28%. The group with a shorter SAD performed better in the 20-cm rise from both feet (*p* < 0.01) (Fig. 4; Table 3).

#### Spinal alignment

Spinal alignment was better in the group with shorter SVA (*p* < 0.001), smaller PT (*p* < 0.001), and larger sacral slope (*p* < 0.05) than in the group with shorter SAD (Table 3).

#### Correlation between VAS score and each movement performance parameter

Correlations between the VAS score and each movement performance parameter were examined, and no significant correlation was found between the VAS score and movement performance in the ES, EL, and LL groups. However, significant differences (*p* < 0.05) were found in the LS group for the two-step, 3-m TUG, and open-eyed one-leg standing time tests (Table 4).

**Table 4:** Correlation results between visual analog scale score and movement performance items

	[ES]		[EL]		[LS]		[LL]	
	r	*P*-value	r	*P*-value	r	*P*-value	r	*P*-value
2 Step	0.02	0.86	-0.03	0.84	-0.28	< 0.05	-0.20	0.07
3 m TUG	0.02	0.90	0.20	0.20	0.24	< 0.05	0.17	0.12
Unipedal standing	-0.03	0.83	-0.09	0.57	-0.24	< 0.05	-0.14	0.20
Hand grip	0.11	0.38	-0.08	0.60	-0.05	0.66	-0.17	0.13

ES group, 65-74-year-old patients with SAD < 160 mm; EL group, 65-74-year-old with SAD ≥ 160 mm;

LS group, ≥ 75-year-old patients with SAD < 160 mm; LL group, ≥ 75-year-old patients with SAD ≥ 160 mm

SAD: abdominal wall-sacral distance, r: correlation coefficient, TUG: timed and up go.

#### Regression analysis

Multiple regression analysis with SAD as the objective variable and age, BMI, waist circumference, CCI, Frailty Index, femur bone mineral density, VAS, SVA, and PT as explanatory variables showed that CCI and waist circumference and SVA and PT were significantly correlated (Table 5).

**Table 5:** Multiple regression analysis results for SAD

Factor	β	SE(β)	stdβ	t-val
CCI (points)	1.90	0.82	0.10	2.31*
Age (years)	0.18	0.17	0.05	1.04
Abdominal circumstance (cm)	1.04	0.16	0.43	6.41**
Frailty Index (points)	-1.23	1.12	-0.05	-1.11
Bone Mineral Density (Hip neck; g/cm^2^)	14.42	12.35	0.05	1.17
BMI (kg/m^2^)	0.89	0.48	0.13	1.84
VAS (lumber)	0.16	0.55	0.01	0.30
SVA (mm)	0.09	0.03	0.17	3.39**
PT (^°^)	0.71	0.12	0.31	6.14**
R	0.78			
RR	0.61			
adjRR	0.60			

SAD: abdominal wall-sacral distance, β: partial regression coefficient, SE(β): standard deviation ofβ, stdβ: standard deviation coefficient, t-val: significance of correlation with objective variable, R: multiple correlation coefficient, RR: coefficient of determination, adjRR: after adjustment for determination factor, CCI: Charlson Comorbidity index, BMI: body mass index, VAS: Visual Analogue Scale, SVA: sagittal vertical axis, PT: pelvic tilt. *P < 0.05, **P < 0.001.

In a logistic regression analysis with SAD ≥ 160 mm as the objective variable and age, BMI, waist circumference, CCI, Frailty Index, femoral bone mineral density, VAS, SVA, and PT as explanatory variables, waist circumference, BMI, femoral bone mineral density, SVA, and PT were the independent factors (Table 6).

**Table 6:** Results of logistic regression analysis with SAD 160 mm or greater as the objective variable

Explanatory variables	β	SE(β)	Odds ratio (95% CI) for SAD more than 160 mm	*P*-value
CCI (points)	-0.13	0.14	0.88 (0.67–1.16)	0.36
Age (years)	-0.02	0.03	0.98(0.93–1.04)	0.52
Abdominal circumstance (cm)	0.07	0.03	1.08(1.02–1.14)	< 0.05
Frailty Index (points)	0.21	0.20	1.23(0.83–1.82)	0.30
Bone Mineral Density (Hip neck; g/cm^2^)	5.30	2.37	205.82(2.00–21234.57)	< 0.05
BMI (kg/m^2^)	0.21	0.09	1.23(1.03–1.467)	< 0.05
VAS (lumber)	0.07	0.09	1.07(0.90–1.29)	0.44
SVA (mm)	0.01	0.00	1.01(1.00–1.02)	< 0.05
PT (^°^)	0.11	0.02	1.12(1.07–1.17)	< 0.001

AIC (Akaike’s information criterion) = 206.43, AUC (area under curve) = 0.90

β: regression coefficients in the model, SE(β): amount of change in odds ratio, SAD: abdominal wall-sacral distance, CCI: Charlson Comorbidity index, BMI: body mass index, VAS: Visual Analogue Scale, SVA: sagittal vertical axis, PT: pelvic tilt.

## Discussion

In this study, we investigated the relationship between SAD and movement performance using SAD measured from simple lateral spine X-ray images. To our knowledge, this was the first attempt to determine how SAD affects movement performance. We used the SAD alone without adjustment for height or weight, similar to the waist circumference measured in the diagnosis of metabolic syndrome, and we plan to investigate further whether the SAD is a new measurement method and should be adjusted for height or weight.

No significant differences were observed in BMI, waist circumference, or trunk fat percentage between the 65–74-year group and the ≥ 75-year group. However, the performance of the ≥ 75-year group was worse than the 65–74-year group in terms of physical activities, such as grip strength, time to stand on one leg with eyes open, 3-m TUG test, and standing up. This can be explained by the decline in movement performance associated with aging [32]. Other reasons might be that the ≥ 75-year group had higher VAS scores in the lumbar region and a higher number of pre-existing vertebral fractures than the 65–74-year group. However, the VAS score differed by only 0.8 on a 10-point scale, and whether this difference could affect movement performance remains unclear. The location and number of vertebral fractures were not examined; however, spinal alignment revealed that the ≥ 75-year group had smaller SS and significantly larger SVA and PT than the 65–74-year group. Anatomically, a larger PT and smaller SS represent a posteriorly tilted pelvis [10]. Kyphotic spine deformity and changes in the posterior tilt of the pelvis may have also affected movement performance.

Therefore, we further subdivided participants in the 65–74-year group and ≥ 75-year group to examine differences in SAD in this study. This allowed for a more detailed analysis of the effects of SAD within the same age group. No significant differences were observed in age and grip strength between the ES and EL and LS and LL groups. Nevertheless, the group with a larger SAD performed significantly worse in terms of the movement performance items, two-step test, 3-m TUG, standing up, and open-eyed one-leg standing time tests than the group with a smaller SAD. Significant differences were also observed in the VAS scores of the lumbar spine between the ES and EL and LS and LL groups, suggesting that pain may have limited the patients’ activities. Among ≥ 75-year group, VAS scores affected the two-steps, TUG, rise from 20 cm, and open-eyed one-leg standing times in the LS group, suggesting that pain was one of the factors limiting movement performance. When VAS scores were correlated with movement performance, no correlation was observed in the ES, EL, and LL groups; however, VAS scores correlated with movement performance in the LS group. Kawaguchi et al. [33] stated that elderly patients with osteoporosis and poor sagittal spine alignment had more vertebral fractures than those with normal spine alignment; they also reported a higher risk of lower back pain and indicated that changes in spinal alignment may have triggered lower back pain.

The rise test was conducted in four steps at 40, 30, 20, and 10 cm. A greater SAD was associated with greater difficulty in rising from lower heights, suggesting that SAD may have influenced the results of the rise test. The differences in the rise test between the ES and EL groups and between the LS and LL groups could be related to differences in spinal alignment (SVA, PT, SS). In a previous study, improvement in postoperative PT contributed to the improvement in open-eyed one-leg standing and TUG times [34], and our study revealed similar results, as a pelvic posterior tilt is likely to decrease activities of daily living (ADL). However, ADL can be maintained if the BMI is high and muscle mass is maintained, even with poor sagittal spine alignment [33]. Maintaining muscle mass is crucial for maintaining movement performance, even in the presence of posterior PT and poor alignment. Considering the definition of SAD, the group with a larger SAD may require a larger support base surface than the group with a smaller SAD because of forward shift of the center of gravity of the body, and the risk of falling was found to be higher in those with a larger SAD. In the future, we plan to investigate the relationship between the total trajectory length of the center of gravity and SAD. In the present study, we investigated what occurs to the 65–74-year old group as they age with their SAD maintaining constant. The results of rising from steps, for the EL and LS groups are similar. These outcomes indicate that if the EL group is > 75 years old and their SAD < 160 mm, they will be similar to the LS group, and their ability to rise from steps will not change significantly. However, if the SAD continues above 160 mm, they will be classified as LL, and their ability to ascend from stairs may be considerably reduced.

Waist circumference is a measurement of the circumference of the torso, which is the sum of the circular distance of the muscles, viscera, subcutaneous fat, and vertebral bones of the torso. In women, the rectus and transversus abdominis muscles represent a significantly larger proportion of the abdominal muscles, allowing them to be representative of the abdominal muscles [35]. Whereas lower movement performance results in higher subcutaneous fat content [36], increasing movement performance can reduce subcutaneous and visceral fat percentage [37], with subcutaneous fat being related to the amount of movement performance. Subcutaneous and visceral fat are also closely related to diet and alcohol intake [38]. SAD is measured excluding subcutaneous fat; therefore, SAD may not be directly related to subcutaneous fat. However, the relationship between SAD and visceral fat should be investigated in future studies.

This study has some limitations. It included patients attending a hospital in an urban area with good public transportation; therefore, the results may differ from those in rural areas. The presence of back pain affected the results of our study; however, we did not investigate whether the patients were taking non-steroidal anti-inflammatory drugs (NSAIDs), analyze the underlying causes of the back pain, or examine their treatment history. Patients with low back pain that were suppressed by NSAIDs may have been included in the study. The examination times were randomized; therefore, diet may have influenced SAD; however, the effect was minimized by limiting the measurement site to below L3, which is lower than the stomach location [39]. No significant differences were observed in the presence or absence of exercise habits; however, exercise intensity was not investigated. In a subgroup analysis of the four groups (ES, EL, LS, and LL) with SAD as the primary outcome variable, 45 patients were required to achieve an alpha error of 0.05 and power of 0.80 [40]; however, the smallest group had 43 patients, and the effect size was small. SAD is a value obtained from a standing radiographic examination, and patients who had difficulty in standing were excluded from the study because of the difficulty in measuring SAD in these patients. This SAD was based on standard BMI cut off values.

## Conclusions

In the present study, SAD correlated with waist circumference and spinal alignment; it was also related to movement performance. In the 65–74-year and ≥ 75-year groups, movement performance items (in terms of the two-step, 3-m TUG, rising from 20 cm, and open-eyed one-leg standing tests) scored better in patients with smaller SAD values. In addition to osteoporosis testing, physical evaluation should be performed in women with osteoporosis aged ≥ 65 years who have high SAD. The SAD is a new potential assessment method, and further research is required to verify its validity and reproducibility.

## Data Availability

The datasets used and/or analyzed in the current study are available from the corresponding authors upon reasonable request.

## Abbreviations

BMI: body mass index
SVA: sagittal vertical axis
PT: pelvic tilt
SS: sacral slope
SAD: sacral vertebra–abdominal wall distance
ES group: 65–74-year-old patients with SAD < 160 mm
EL group: 65–74-year-old patients with SAD ≥ 160 mm
LS group: ≥ 75-year-old patients with SAD < 160 mm
LL group: ≥ 75-year-old patients with SAD ≥ 160 mm
VAS: visual analog scale
ADL: activities of daily living
SD: standard deviations
TUG: Timed Up and Go
CCI: Charlson Comorbidity Index
