# Correction: Relationship between sacral-abdominal wall distance, movement performance, and spinal alignment in osteoporosis: a retrospective study

**DOI:** 10.1186/s12877-024-04908-3

**Published:** 2024-05-02

**Authors:** Takashi Nagai, Makoto Miyagami, Shota Nakamura, Keizo Sakamoto, Koji Ishikawa, Ichiro Okano, Fumihito Kasai, Yoshifumi Kudo, Nobuyuki Kawate

**Affiliations:** 1 Department of Rehabilitation Medicine, Showa University School of Medicine, 1-5-8 Hatanodai, Shinagawa-ku, 142-8666 Tokyo, Japan; 2 Orthopaedic Surgery, Showa University School of Medicine, 1-5-8 Hatanodai, Shinagawa-ku, 142-8666 Tokyo, Japan

**Correction: BMC Geriatrics (2024) 24:252**

10.1186/s12877-024-04865-x

After publication of this article, the authors reported that

- in the pdf file, in Tables 3 and 4, the headers should have been written with brackets (e.g., [ES]);

- in Tables 5 and 6, the symbol ‘˚’ should have been superscript (instead of centered).

The original article has been corrected.

